# Prohibitin: A Novel Molecular Player in KDEL Receptor Signalling

**DOI:** 10.1155/2015/319454

**Published:** 2015-05-10

**Authors:** Monica Giannotta, Giorgia Fragassi, Antonio Tamburro, Capone Vanessa, Alberto Luini, Michele Sallese

**Affiliations:** ^1^Department of Cellular and Translational Pharmacology, Fondazione Mario Negri Sud, 66030 Santa Maria Imbaro, Italy; ^2^Department of Cell Biology & Signalling, The FIRC Institute of Molecular Oncology (IFOM), 20139 Milan, Italy; ^3^Proteomic Core Facility, Fondazione Mario Negri Sud, 66030 Santa Maria Imbaro, Italy; ^4^Institute of Protein Biochemistry, National Research Council and Telethon Institute of Genetics and Medicine, 80131 Naples, Italy

## Abstract

The KDEL receptor (KDELR) is a seven-transmembrane-domain protein involved in retrograde transport of protein chaperones from the Golgi complex to the endoplasmic reticulum. Our recent findings have shown that the Golgi-localised KDELR acts as a functional G-protein-coupled receptor by binding to and activating Gs and Gq. These G proteins induce activation of PKA and Src and regulate retrograde and anterograde Golgi trafficking. Here we used an integrated coimmunoprecipitation and mass spectrometry approach to identify prohibitin-1 (PHB) as a KDELR interactor. PHB is a multifunctional protein that is involved in signal transduction, cell-cycle control, and stabilisation of mitochondrial proteins. We provide evidence that depletion of PHB induces intense membrane-trafficking activity at the ER–Golgi interface, as revealed by formation of GM130-positive Golgi tubules, and recruitment of p115, *β*-COP, and GBF1 to the Golgi complex. There is also massive recruitment of SEC31 to endoplasmic-reticulum exit sites. Furthermore, absence of PHB decreases the levels of the Golgi-localised KDELR, thus preventing KDELR-dependent activation of Golgi-Src and inhibiting Golgi-to-plasma-membrane transport of VSVG. We propose a model whereby in analogy to previous findings (e.g., the RAS-RAF signalling pathway), PHB can act as a signalling scaffold protein to assist in KDELR-dependent Src activation.

## 1. Introduction

Intracellular organelles maintain their homeostasis through the continuous exchange of proteins and lipids, which tend to intermix during membrane trafficking. The transport of cargoes from the endoplasmic reticulum (ER) to the Golgi complex involves temporary mislocalisation of ER-resident proteins into post-ER compartments. By virtue of their KDEL sequence, these ER proteins can bind to the KDEL receptor (KDELR) and are thence shuttled back to the ER. To date, three different genes have been identified in human that encode the closely related KDELRs: KDELR1, KDELR2, and KDELR3 [1–4].

The KDELR is an integral membrane protein with seven transmembrane domains [5]. The N-terminal of the receptor faces the lumen of the organelles, while the C-terminal is in the cytosol [6, 7]. In addition to the rescue of chaperones, the KDELR takes part in the regulation of the ER stress response, by modulation of the p38 mitogen-activated protein kinases (MAPKs) [8]. The KDELR also activates extracellular signal-regulated kinases (ERKs) and autophagy, which contributes to the clearance of intracellularly aggregated mutant proteins, such as superoxide dismutase 1 (SOD1), *α*-synuclein, and the pathological huntingtin [9]. Recently, we identified a novel signalling cascade that is activated by the KDELR at the Golgi complex [10, 11]. Here, the KDELR acts as a G-protein-coupled receptor (GPCR) and stimulates Gq, which, in turn, promotes the activation of the Src family kinases (SFKs) [10, 11]. These active SFKs trigger a complex tyrosine phosphorylation cascade that controls membrane trafficking from the Golgi to the plasma membrane [10].

In the present study, while looking for novel KDELR interactors that might participate in the signal transduction of the KDELR, we identified prohibitin-1 (PHB). PHB is a member of an evolutionarily conserved family of proteins that includes PHB-2, stomatin, erlins, flotillins, and the bacterial protein HflK. These proteins share the stomatin/prohibitin/flotillin/HflK/C (SPFH) domain, which is also known as the PHB domain [12]. PHB-domain proteins are integral membrane proteins or are strongly associated with cell membranes via posttranslational modifications (i.e., acyl moieties) or via hydrophobic regions. These proteins have the tendency to oligomerise and to segregate into lipid rafts, which are specific membrane subdomains that are enriched in cholesterol and glycosphingolipids [12]. PHB-domain proteins are widely distributed in the different cellular organelles, including the mitochondria, the ER, the Golgi complex, the endosomes, and the plasma membrane [13–16].

PHB is a 30-kDa protein with a single membrane spanning domain mainly localised in the inner mitochondrial membrane, where it functions as a chaperone and controls proteostasis of mitochondrial proteins [17, 18]. PHB knock-down affects the organisation of the mitochondrial network, possibly by preventing membrane fusion, although it does not alter the mitochondrial membrane potential and the ATP generation system [13, 19]. In addition to mitochondria, PHB is in the nucleus, where it modulates DNA transcription, and in the plasma membrane, where it modulates receptor signalling downstream of the insulin receptor and protease-activated receptor 1 (PAR1) [13, 16, 20]. Insulin promotes PHB phosphorylation on Y114 and Y259, and via Akt on T258 [21, 22]. These phosphorylations are required for the adaptor functions of PHB on RAS-dependent activation of RAF1/ERK signalling [16]. PHB carries out these tasks by physical association with AKT and RAF1. From a cell-biology standpoint, PHB can promote cancer cell growth and formation of metastasis in animal models [16, 19, 23]. Furthermore, the levels of PHB on the plasma membrane correlate with cancer outcome, highlighting the importance of PHB in cancer signalling [16].

In the present study, we have identified a KDELR–PHB complex and explored the functional role of PHB along the KDELR signalling pathway, as well as in its coordination of membrane trafficking. We provided evidence that PHB is required for KDELR-dependent SFKs activation, transport of cargo from the Golgi complex to the plasma membrane, and membrane trafficking between the Golgi complex and the ER.

## 2. Results

### 2.1. Signalling and Membrane Transport Machinery Proteins Coimmunoprecipitate with the KDELR

To elucidate the molecular events triggered by the KDELR in the regulation of membrane trafficking, we used a coimmunoprecipitation approach. The best antibody available against the endogenous KDELR recognizes an epitope sequence at the KDELR C-tail. As this region is believed to be involved in binding with KDELR interactors, to prevent any bias in our immunoprecipitation, we used a transfected epitope-tagged version of the KDELR [11]. Furthermore, as transfection efficiency can vary across different experiments, we decided to exploit HeLa cells stably transfected with myc-tagged KDELR (HeLa-myc cells), which were previously used to study interactions between the KDELR and ARF GAP1 [24]. Therefore, these HeLa-myc cells represent a good tool to investigate KDELR interactors.

Initially, we characterised this cell line to determine whether this stable transfection of the myc-tagged KDELR affects distribution of the endogenous KDELR, Golgi complex organisation, or membrane transport. Here, the intracellular distribution of the stably transfected myc-tagged KDELR was similar to that of endogenous KDELR, and their expression levels were also comparable (see Supplementary Figure S1 in Supplementary Material available online at http://dx.doi.org/10.1155/2015/319454). Also, the structure of the Golgi complex was not affected in the HeLa-myc cells, as assessed by GM130, mannosidase II and TGN46 staining (Supplementary Figure S1). The membrane-traffic efficiency was investigated using the temperature-sensitive mutant vesicular stomatitis virus G glycoprotein (VSVG). This mutant VSVG is synchronisable according to temperature, and it has been widely used to assess protein folding and the efficiency of the secretory pathway [10]. At 40°C, VSVG cannot fold completely in the ER, and consequently its exit from the ER is blocked (temperature-block). When the cells are then shifted to 32°C (temperature-block release), VSVG can fold and leave the ER, and in this way it is synchronously transported through the secretory pathway to the plasma membrane. The transport kinetics of VSVG in HeLa-myc cells was similar to that in wild-type HeLa cells (Supplementary Figure S2). In view of these data, we considered that the HeLa-myc cells represent a suitable model for coimmunoprecipitation experiments.

To determine whether the immunoprecipitation conditions were appropriate for coimmunoprecipitation of the KDELR with its interacting partners, we searched for proteins that should interact with the KDELR on the bases of previous studies, or that are components of the retrograde transport machinery. The HeLa-myc and control HeLa cells were lysed, and the proteins were immunoprecipitated using agarose-conjugated anti-c-myc antibodies. The immunoprecipitated proteins were analysed by Western blotting, which revealed the Gs and Gq subunits of the heterotrimeric G proteins [11, 25], and the *β* subunit of the COPI coatomer complex (Supplementary Figure S3). These proteins were absent in the immunoprecipitate obtained from the wild-type control HeLa cell lines. These data indicated that the chosen cell model and experimental conditions are optimal to reveal KDELR interactors.

### 2.2. Identification of KDELR Interactors by Mass Spectrometry

To identify KDELR interactors, we carried out preparative coimmunoprecipitation of the KDELR from the control and HeLa-myc cells. The immunoprecipitated proteins were separated by two-dimensional gel electrophoresis, and then the gels were silver stained. Differentially immunoprecipitated proteins were excised, subjected to tryptic digestion, and analysed by matrix-assisted laser desorption/ionisation time-of-flight mass spectrometry (MALDI-TOF MS). The peptide masses obtained were matched to peptide mass databases using the ProFound and MASCOT software. Peptide matching and protein searches were performed by submitting the peptide mass lists to database searches on NCBInr and/or SWISS PROT, using the MASCOT and ProFound search engines.

This analysis identified PHB as a potential KDELR interactor. In view of our interest in the signalling functions of the KDELR, PHB was further investigated.

### 2.3. PHB Coimmunoprecipitates with the KDELR

**Figure 1:**
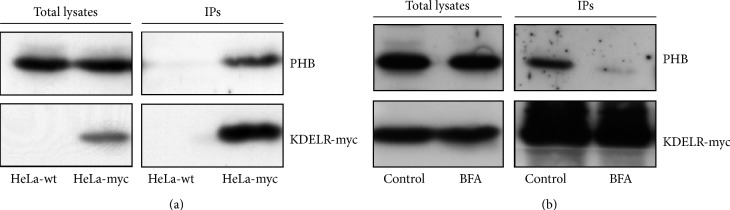
The KDELR–PHB interaction. (a) PHB coimmunoprecipitates with the KDELR. Protein from wild-type (HeLa-wt, control) and stably transfected KDELR-myc (HeLa-myc) HeLa cells were immunoprecipitated using anti-myc antibodies. The proteins from cell lysates (Total lysates) and immunoprecipitated (IPs) were separated by polyacrylamide gel electrophoresis and analysed by Western blotting for PHB and KDELR-myc. The images shown are representative of three independent experiments. (b) BFA treatment dissociates the KDELR–PHB complex. HeLa-myc cells were treated with vehicle (Control) or for 5 min with 5 *μ*g/mL BFA and the proteins were immunoprecipitated with anti-myc antibodies. The proteins from cell lysates (Total lysates) and immunoprecipitated (IPs) were separated by polyacrylamide gel electrophoresis and analysed by Western blotting for PHB and KDELR-myc, as indicated. The images shown are representative of two independent experiments.

To confirm that PHB is part of the KDELR interactome, HeLa-myc cells stably expressing the KDELR-myc chimera were subjected to coimmunoprecipitation using an agarose-conjugated anti-c-myc antibody, followed by Western blotting with an anti-PHB antibody. This approach confirmed coprecipitation of PHB with the KDELR (Figure 1(a)). To exclude potential non-specific binding of PHB with the agarose resin, we performed the same immunoprecipitation from lysates of wild-type HeLa cells. Here, the PHB protein did not show any intrinsic interactions with the agarose-conjugated anti-c-myc antibody (Figure 1(a)).

To further understand the KDELR–PHB association, we modulated the KDELR interaction with PHB by perturbing Golgi homeostasis using brefeldin A (BFA) [26]. The fungal metabolite BFA induces rapid and reversible disassembly of the Golgi stack into tubules and vesicles, which results in redistribution of the majority of Golgi membranes and enzymes into the ER, in a reversible manner. BFA treatment results in redistribution of the KDELR into a remnant of the ER–Golgi intermediate compartment. In this context, we examined whether this redistribution of the KDELR that is induced by BFA treatment affects the interaction of the KDELR with PHB. HeLa-myc cells were treated with 5 *μ*g/mL BFA for 5 min and then subjected to coimmunoprecipitation using an agarose-conjugated anti-c-myc antibody, which was followed by Western blotting with an anti-PHB antibody. After this 5 min of BFA treatment the KDELR lost its interaction with PHB (Figure 1(b)). These data strengthen the specificity of this interaction between the KDELR and PHB. In addition, since BFA targets the Sec7-type GTP-exchange factors for ARF1, the KDELR–PHB interaction might be regulated by ARF.

**Figure 2:**
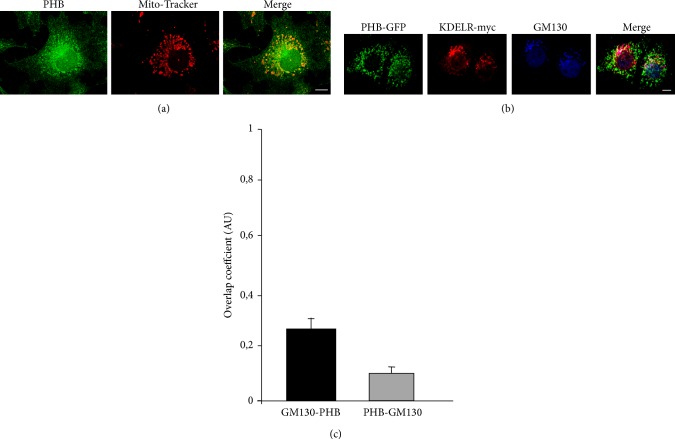
Intracellular distribution of PHB. (a) PHB mainly localises to the mitochondria. COS-7 cells were fixed, permeabilised, and stained for PHB (green) and Mito-Tracker (red). The merged image of green and red signals is also shown. (b) Colocalisation analysis of PHB and Golgi proteins. COS-7 cells were transiently transfected with GFP-tagged PHB and myc-tagged KDELR. The day after, the cells were fixed and stained for myc (red), and GM130 (blue). The merged image of the green (PHB-GFP), red and blue signals is also shown. (a, b) The images shown are representative of three independent experiments. Scale bars, 10 *μ*m. (c) Quantification of PHB-GFP fluorescence colocalizing with GM130. The black bar indicates the extent of GM130 overlapping with PHB. The gray bar indicates the extent of PHB overlapping with GM130. Data are means of overlapping coefficient ± SEM, representative of two independent experiments assessing at least 25 cells each. AU: arbitrary units.

Previous studies have demonstrated that PHB is mainly localised to the mitochondria, although it has also been reported to localise to the nucleus and the plasma membrane [13, 16, 17, 27]. To support our coprecipitation data, we investigated whether minor amounts of PHB can localise to the Golgi complex. Endogenous PHB was labelled in COS-7 cells and counterstained with the mitochondrial marker Mito-Tracker (Figure 2(a)) or GM130 as a Golgi marker (not shown). We confirmed that the majority of PHB was in the mitochondria, although the plasma membrane was also labelled, while it was difficult to reveal PHB on the Golgi complex. To better understand the relationships of PHB labelling with the KDELR and the Golgi complex, COS-7 cells were transfected with GFP-tagged PHB and myc-tagged KDELR. A minor fraction of PHB colocalised with the* cis*-Golgi marker GM130 and KDELR-GFP, indicating that the Golgi membranes can be targeted by PHB (Figures 2(b) and 2(c)).

### 2.4. Functional Morphology of the Secretory Pathway in PHB-Depleted Cells

**Figure 3:**
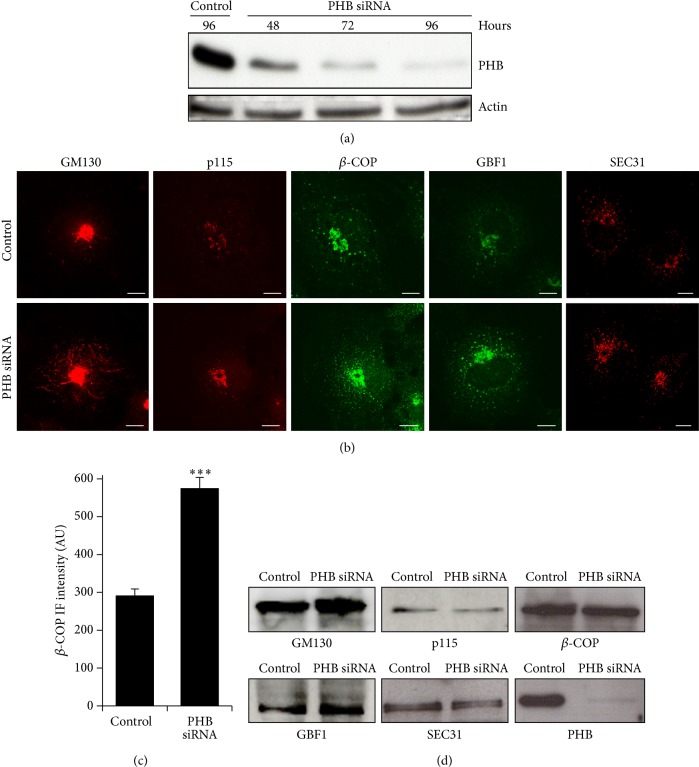
Effects of PHB knock-down on five proteins localised at the ER–Golgi interface. (a) Analysis of PHB knockdown efficiency. HeLa-myc cells were treated with siRNAs against PHB (PHB siRNA) for the indicated times. Scrambled interfered cells (Control) are shown as reference. The cells were lysed, and the proteins were analysed by Western blotting for PHB expression levels. Actin was used as the loading control. (b) PHB knockdown affects the intracellular distribution of ER and Golgi proteins. Mock-interfered (Control) and PHB-interfered (PHB siRNA) COS-7 cells were fixed and stained for GM130, p115, *β* subunit of the COPI coatomer complex (*β*-COP), GBF1 and SEC31, as indicated. The images are representative of two independent experiments. Scale bars, 10 *μ*m. (c) Quantification of *β*-COP immunofluorescence levels on the Golgi complex. Data are means ± SEM of *β*-COP immunofluorescence from two independent experiments, with at least 25 cells quantified per experiment. ^∗∗∗^ *p* < 0.001 compared to control cells (*t*-test). AU: arbitrary units. (d) PHB knockdown does not affect the expression levels of ER and Golgi proteins. COS-7 cells treated as in B were lysed and their proteins analysed by Western blotting using antibodies to GM130, p115, *β*-COP, GBF1 and SEC31. The levels of PHB knockdown were investigated as a control.

To gain insight into the role of PHB at the Golgi complex, we carried out morphological analysis of the secretory pathway in cells under RNA interference for PHB. HeLa-myc cells were treated with PHB small-interfering (si)RNA, and PHB expression was analysed at various times. PHB was down-regulated by 70% already after 48 h of this RNA interference, and reached about 90% knock-down after 96 h (Figure 3(a)). This later time (i.e., 96 h) was used for the morphological analysis. These PHB-interfered HeLa-myc cells were labelled for five key proteins that are localised at the ER–Golgi interface and are involved in anterograde and retrograde membrane trafficking. The Golgi matrix protein GM130 localises to the* cis*-Golgi. It is involved in Golgi stacking and membrane trafficking via tubular membranes from the* cis*-Golgi to the most distal region of the ER–Golgi intermediate compartment. The vesicle-tethering protein p115 is a GM130-interacting protein that is localised to the* cis*-Golgi and is involved in the transport of cargoes from the ER to the* cis*-Golgi. The coatomer protein complex-I (COPI) is a protein complex that coats newly forming membrane carriers (vesicles, tubules), and participates in intra-Golgi transport and retrograde Golgi-to-ER transport. *β*-COP is a component of the COPI complex, and it has been used to monitor the activity and distribution of COPI. Golgi BFA-resistant guanine nucleotide exchange factor 1 GBF1 is an activator of the small GTPase ARF and is also a part of the COPI complex. COPII is a protein complex that coats newly forming membrane carriers (vesicles and tubules) that exit the ER. SEC31 is a component of COPII, and it has been used to monitor the activity and distribution of COPII.

Remarkably, the knock-down of PHB induced strong changes in the intracellular distribution of these markers, as compared to control (mock) cells (Figure 3(b)). Specifically, GM130 showed intense tubulation in about 25% of the interfered cells that might be indicative of a strong trafficking activity at the Golgi interface (Figure 3(b)). The amount of p115 recruited to the Golgi complex was increased, which also suggested stronger membrane trafficking activity (Figure 3(b)). *β*-COP immunofluorescence at the Golgi as well as on peripheral dots (i.e., transport carriers) was also increased (Figures 3(b) and 3(c)). GBF1 showed stronger staining in the central Golgi area, as well as of peripheral transport carriers, which were more numerous (Figure 3(b)). Similarly, there were many more SEC31-positive carriers, and these accumulated towards the central Golgi area (Figure 3(b)). Finally, we examined the expression levels of these proteins in control and PHB interfered cells. As shown in Figure 3(d) the amounts of GM130, p115, *β*-COP, GBF1 and SEC31 were not substantially affected by the knockdown of PHB indicating that the increased immunofluorescent staining is caused by an activation/recruitment of these proteins to the membranes.

Altogether, these findings suggest that removal of PHB stimulates membrane trafficking between the Golgi and the ER.

### 2.5. Knock-Down of PHB Interferes with KDELR-Dependent SFK Activation

Our group reported that KDELR stimulation triggers a signalling cascade that activates SFKs and controls membrane trafficking [10]. Here, we hypothesise a model where in an analogy with previous findings (the RAS-RAF pathway [19]), PHB can act as a signalling scaffold protein and assist in KDELR-dependent SFKs activation. To test this hypothesis, we determined the activation of the SFKs in PHB knocked-down cells.

**Figure 4:**
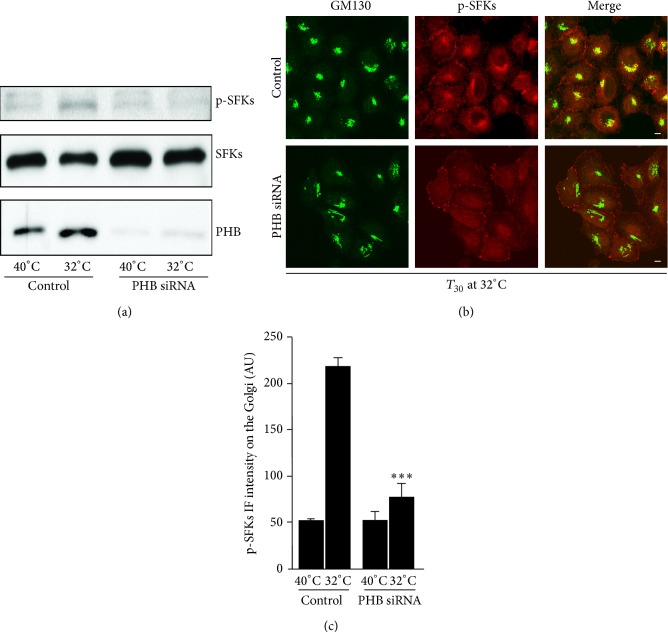
PHB knock-down hinders KDELR-dependent SFKs activation. (a) Depletion of PHB inhibits SFKs activation. HeLa-myc cells were treated with scrambled siRNAs (Control) or siRNAs against PHB (PHB siRNA) for 96 h. After infection with VSV for 45 min, the cells were incubated at 40°C for 3 h (temperature block) and then shifted to 32°C for 30 min (block release). The cells were lysed and analysed by Western blotting for active phosphorylated SFKs (p-SFKs). The total SFKs (SFKs) was used as the loading control, while the knock-down levels were assessed with a PHB antibody. The images shown are representative of two independent experiments. (b) Depletion of PHB inhibits SFKs activation on the Golgi complex. HeLa-myc cells were treated as in (a). Following the 30 min of temperature-block release, the control cells and siRNA-treated cells were fixed and stained for GM130 (marker for Golgi area definition; green) and for active SFKs (p-SFKs; red); the merged images are also shown. Scale bars, 10 *μ*m. (c) Quantification of p-SFKs immunofluorescence intensity on the Golgi complex of HeLa-myc cells treated as in (a). Data are means ± SEM of three independent experiments, with at least 50 cells quantified per experiment. ^∗∗∗^ *p* < 0.001 compared to control cells at 32°C (*t*-test).

Our previous study showed that a transport pulse of VSVG activates the KDELR, and in turn, the SFKs [10]. Thus, PHB knocked-down HeLa-myc cells were infected with VSV, exposed to a pulse of VSVG traffic [10], and analysed for the activation of SFKs using Western blotting. The SFKs were activated in the controls and the mock-interfered cells, while their activation was prevented in the PHB knock-down cells (Figure 4(a)). In addition, with activation of the SFKs monitored by confocal immunofluorescence analysis, this confirmed strong inhibition of SFKs activation at the Golgi complex (Figures 4(b) and 4(c)). This suggested that PHB is required for activation of the SFKs downstream of the traffic-pulse-dependent KDELR signalling cascade.

According to our published model, cargo proteins are transported from the ER to the Golgi complex together with KDEL proteins. These KDEL proteins can then bind to the KDELR, and activate the signalling cascade and the SFKs [10]. Thus, any impairment of the ER-to-Golgi traffic step might prevent SFKs activation.

**Figure 5:**
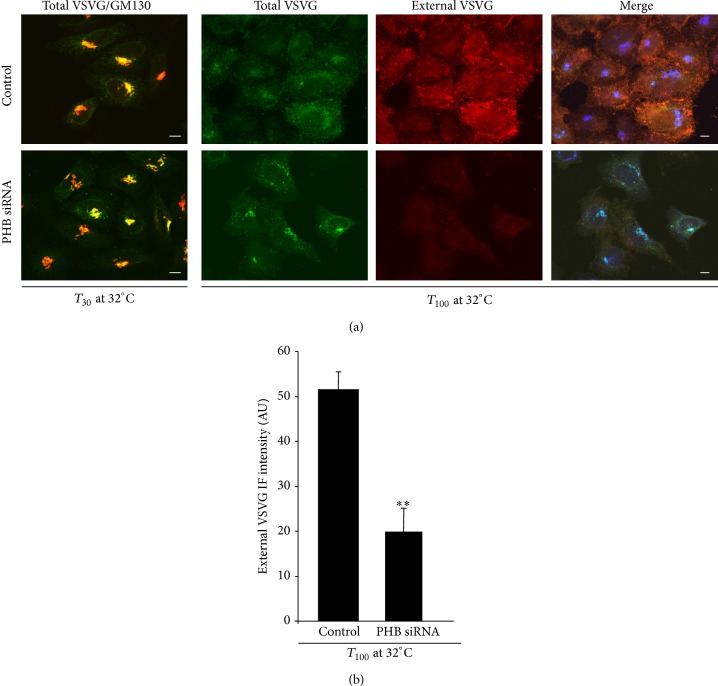
Trafficking of VSVG in PHB knock-down cells. (a) Depletion of PHB does not interfere with transport of VSVG to the Golgi complex, but it impairs VSVG arrival at the plasma membrane. HeLa-myc cells were treated with scrambled siRNAs (Control) or with siRNAs against PHB (PHB siRNA,) for 96 h. After infection with VSV for 45 min, the cells were incubated at 40°C for 3 h (temperature block) and then shifted to 32°C for the indicated times (temperature-block release). Left panels: The cells were fixed and stained for VSVG (green) and GM130 (marker for Golgi area definition, red); the merged images are also shown (Total VSVG/GM130). Right panels: Immunostaining for total VSVG (green), VSVG at the plasma membrane (revealed by an antibody against the extracellular domain of VSVG; External VSVG, red). The merged images shown include immunostaining for GM130 (blue). Scale bars, 10 *μ*m. (b) Quantification of VSVG localised at the plasma membrane of HeLa-myc cells treated as in (a). Data are means ± SEM of VSVG immunofluorescence on the plasma membrane, from three independent experiments, with at least 50 cells quantified per experiment. ^∗∗^ *p* < 0.05 compared to control cells (*t*-test).

To determine whether the SFKs activation and inhibition observed in PHB-depleted cells is caused by a transport problem, we monitored VSVG transport from the ER to the Golgi complex. Indeed, VSVG was efficiently transported to the Golgi complex in PHB-interfered HeLa-myc cells (Figure 5(a)).

### 2.6. Knock-Down of PHB Interferes with Golgi-to-Plasma-Membrane Transport of VSVG

SFKs inhibition results in the accumulation of VSVG in the Golgi complex, which prevents its arrival at the plasma membrane [10]. Here, we anticipated that in agreement with our previous data, by preventing SFKs activation, PHB knock-down would impair Golgi-to-plasma-membrane transport of VSVG.

HeLa-Myc cells with PHB knocked down were infected with VSV, and transport of VSVG was monitored 30 min and 100 min after the release of the traffic block. At 30 min, in the control (mock interfered) and PHB-interfered cells, VSVG was mainly localised in the Golgi complex. At 100 min, VSVG had reached the plasma membrane in the controls, whereas it remained in the Golgi complex in PHB-interfered cells (Figure 5). This indicated that PHB participation in the KDELR–SFKs signalling pathway is also required for efficient Golgi-to-plasma-membrane transport.

### 2.7. PHB Depletion Promotes Retrograde Golgi-to-ER Transport of the KDELR

ER-resident proteins bearing the KDEL motif can reach post-ER compartments during membrane trafficking [28–32]. The KDELR cycles between the Golgi complex and the ER, and it binds and shuttles these proteins back to the ER. This triggers the KDELR signalling cascade. Thus any perturbation of the KDELR cycling might reduce the amount of KDELR in the Golgi compartment, and thus affect KDELR-mediated SFKs activation.

**Figure 6:**
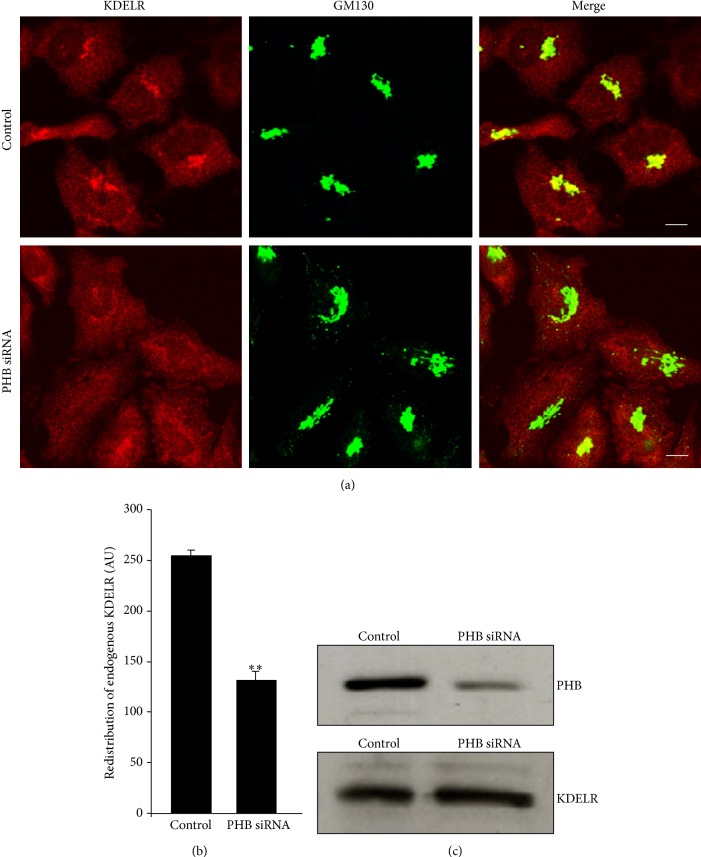
Intracellular distribution of endogenous KDELR in PHB knock-down cells. (a) KDELR redistributes from the Golgi in PHB-depleted cells. HeLa cells were treated with scrambled siRNAs (Control) or with siRNAs against PHB (PHB siRNA,) for 96 h. The cells were fixed and stained for KDELR (red) and GM130 (marker for Golgi area definition, green); the merged images are also shown. Scale bars, 10 *μ*m. (b) Quantification of KDELR in the Golgi area of HeLa cells treated as in (a). Data are means ± SEM for KDELR immunofluorescence on the Golgi, from three independent experiments, with at least 50 cells quantified per experiment. ^∗∗∗^ *p* < 0.001 compared to control cells (*t*-test). (c) PHB knockdown does not affect the expression levels of KDELR. HeLa cells treated as in (a) were lysed, and their proteins analysed by Western blotting using antibodies to KDELR. The levels of PHB knockdown were investigated as a control.

To understand how PHB knock-down might hinder KDELR–SFKs activation, we investigated the distribution of the KDELR in PHB-interfered HeLa cells. Immunofluorescence analysis of the endogenous KDELR showed major amounts in the Golgi complex and lesser amounts in the ER. PHB-depleted cells showed a striking reduction in the KDELR in the Golgi complex (Figures 6(a) and 6(b)). Western blot analysis of the KDELR ruled out the possibility that the low levels of Golgi-KDELR observed in PHB knockdown cells might be caused by a reduced KDELR expression or an increased degradation (Figure 6(c)).

**Figure 7:**
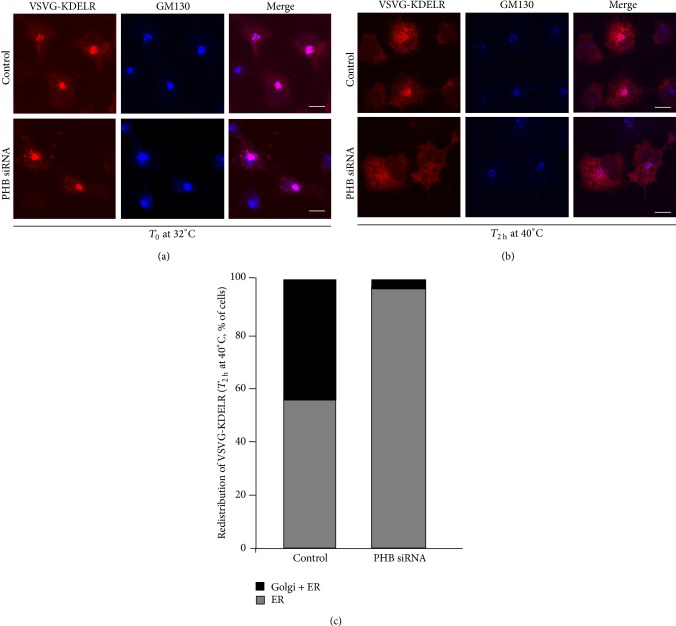
The intracellular dynamics of VSVG-KDELR chimera are affected by PHB knock-down. (a, b) The VSVG–KDELR chimera redistributes towards the ER in PHB-depleted cells. COS-7 cells were treated with scrambled siRNAs (Control) and with siRNAs against PHB (PHB siRNA,) for 48 h, transfected for the VSVG–KDELR chimera, incubated overnight at 32°C, and fixed (a), or following the incubation at 32°C, the cells were further incubated at 40°C for 2 h, and fixed (b). The cells were stained for the VSVG–KDELR chimera (red) and GM130 (marker for Golgi area definition, blue); the merged images are also shown. Scale bars, 10 *μ*m. (c) Quantification of the KDELR in the Golgi and ER, or exclusively in the ER of COS-7 cells treated as in (b). Data are means ± SEM of three independent experiments, with at least 50 cells quantified per experiment. ^∗∗^ *p* < 0.05 compared to control cells (*t*-test).

These reduced levels of endogenous KDELR in the Golgi complex of PHB-depleted HeLa cells might be caused by an imbalance in the cycling of the KDELR between the ER and the Golgi complex. We investigated this possibility using an artificial chimeric protein constituted by the luminal domain of VSVG linked to the N-terminus of the KDELR (VSVG–KDELR). Previous studies demonstrated that VSVG–KDELR accumulates in the Golgi complex when cells are incubated at 32°C, while at 40°C, VSVG–KDELR accumulates in the ER. Shifting the temperature of incubation of the cells from 32°C to 40°C, it is possible to follow retrograde transport of the KDELR from the Golgi to the ER. PHB-interfered COS-7 cells were transfected with the VSVG–KDELR chimera and incubated overnight at 32°C. The day after, the cells were incubated at 40°C for 2 h, and the localisation of the chimera was analysed by confocal microscopy. VSVG–KDELR accumulated in the Golgi complex of controls and PHB-interfered cells upon incubation at 32°C (Figure 7(a), upper panels). Two hours after the shift to 40°C, in 90% of the PHB-interfered cells, the VSVG–KDELR chimera was relocated to the ER, while only 60% of the controls showed this phenotype (Figure 7(a), lower panels, Figure 7(b)).

This all indicates that PHB depletion affects KDELR homeostasis by accelerating its retrograde movement from the Golgi to the ER. The reduced levels of KDELR at the Golgi complex can contribute to decreased KDELR-dependent activation of SFKs in PHB knock-down cells.

## 3. Discussion

The KDELR belongs to the PQ-loop protein family [33], which is distantly related to the GPCR superfamily [34, 35], and resembles the GPCRs in topology and folds of its transmembrane helices [34, 35]. KDELR-bound chaperones [5] activate a Golgi pool of the heterotrimeric G proteins Gq and Gs. Gs activates a signalling cascade at the* cis*-Golgi, which results in activation of retrograde membrane transport [25], while Gq acts by inducing the activation of the SFKs, which phosphorylate a number of proteins, and allows anterograde traffic [10].

In the present study, to uncover the molecular players involved in the KDELR signal-transduction machinery, we searched for interactors of the KDELR using a coimmunoprecipitation and mass spectrometry approach. We identified PHB among the proteins that coimmunoprecipitated with the KDELR from HeLa cells. The KDELR–PHB complex was validated by modulation of their interactions upon BFA treatment. BFA inhibits the guanine-nucleotide exchange factors on ADP-ribosylation factor (ARF-GEFs), thus resulting in inactivation of ARF and loss of the KDELR–PHB interaction. This suggests that the KDELR–PHB complex relies on a functional ARF1.

To date, intracellular PHB has been reported in the mitochondria and nucleus and at the plasma membrane but not in the ER or the Golgi complex, while the KDELR cycles between the ER and the Golgi [13, 29]. In the present study we provided evidences that a minor fraction of PHB can localise to the Golgi complex.

However, to better support this novel relationship between the KDELR and PHB, we carried out a database analysis, with a search for any PHB interactors that have a well-established localisation to or function in the ER or the Golgi complex. Remarkably, PHB interacts genetically with the following yeast proteins: ARV1, an integral membrane protein that cycles between the ER and the Golgi complex and is involved in sphingolipid transport [36]; ERG5, a desaturase that is resident in the ER and is involved in ergosterol biosynthesis [36]; MMM1, which is an integral component of the ER membrane and part of the ER–mitochondria encounter structure (ERMES) [37]; SAC1, which is a phosphatidylinositol phosphate phosphatase that is an integral membrane protein and that cycles between the ER and the Golgi complex, to regulate protein trafficking [38]; and SCT1, which is an integral ER-membrane protein with a glycerol 3-phosphate/dihydroxyacetone phosphate acyltransferase activity [39]. Note that SCT1 interacts genetically with several ER-to-Golgi trafficking proteins, including the GET complex. The GET complex interacts with the yeast HDEL receptor (the yeast orthologue of the mammalian KDELR) in the retrieval of ER chaperones from the Golgi complex to the ER [40]. These genetic interaction data were obtained from the* Saccharomyces* Genome Database (http://www.yeastgenome.org/).

Furthermore, the yeast PHB protein has been detected in complexes with: ERP1, which is a member of the p24 family proteins and a component of the KDELR functional machinery [41]; MNN9, MNN10 and MNN11, which are components of the Golgi mannosyltransferase complex [42]; ANP1, which is an integral membrane protein of the* cis*-Golgi and a component of the alpha-1,6 mannosyltransferase complex [42]; and PMR1, which is an integral P-type ATPase of the Golgi membrane that can transport Ca^2+^/Mn^2+^ ions into the Golgi complex [43]. These data were also obtained from the* Saccharomyces* Genome Database (http://www.yeastgenome.org/). Finally, the *α* subunit of the human COPI complex has been identified in a complex with PHB (http://bioinfow.dep.usal.es/apid/index.htm).

These numerous relationships among PHB and components of the ER and Golgi include proteins that are strictly related to the KDELR (e.g., p24 proteins and COPI subunits), and they strongly support the presence of PHB in these organelles, and its functional relationship with the KDELR.

PHB has an important role in the signalling pathway triggered by the RAS oncogene [13]. Indeed, PHB is required for the activation of RAF1 kinase downstream of RAS, and thus for cell growth [13]. According to the common model, PHB acts as a scaffold to productively direct the RAS–RAF1 interaction [19]. Remarkably, rocaglamides that target PHB impair the RAS–RAF1 interaction, and consequently prevent cancer-cell growth [44]. Furthermore, a recent study has demonstrated that PHB interacts and regulates the localisation and signalling of the PAR1 GPCR [20].

Here, we have reported that the knock-down of PHB mislocalises the KDELR (a functional GPCR [11]) and inhibits KDELR signalling, as for RAS–RAF signalling. In analogy to previous findings, we suggest that PHB acts as a scaffold to retain the KDELR in the appropriate* cis*-Golgi location and to allow activation of the SFKs.

Finally, in agreement with our previous data that showed that inhibition of KDELR-SFKs signalling impairs the transport of VSVG from the Golgi complex to the plasma membrane [10], the present study indicates that PHB knock-down interferes with KDELR–SFK activation thus leading to VSVG accumulation in the Golgi complex, which prevents it from reaching the plasma membrane.

## 4. Materials and Methods

### 4.1. Antibodies

The following antibodies were used: rabbit anti-c-myc polyclonal (Santa Cruz); rabbit anti-PHB polyclonal (NeoMarkers, Fremont CA, USA); mouse anti-*β*-COP monoclonal (Affinity BioReagents, Golden CO, USA); rabbit anti-lysozyme polyclonal (Chemicon); rabbit anti-p-SFKs polyclonal (p-Tyr^418^) (BioSource, CA, USA); rabbit anti-SFKs polyclonal (Santa Cruz Biotechnology); mouse anti-GM130 monoclonal and mouse anti-GBF1 monoclonal (Transduction Laboratories, Lexington, KY, USA); the mouse P5D4 anti-VSVG monoclonal (Sigma Aldrich); and rabbit VSVG luminal domain polyclonal (A. De Matteis), Rabbit p115 [45] and SEC31 were kindly provided by G. Di Tullio. Secondary antibodies were Alexa 488-, Alexa 546- (Molecular Probes, OR, USA), and Cy3-conjugated (Sigma-Aldrich).

### 4.2. Cell Handling and Transport Synchronization Protocols

*Cell Handling*. Human HeLa cells were maintained in Dulbecco's modified Eagle's medium supplemented with 10% foetal calf serum, 2 mM L-glutamine, 100 *μ*g/mL streptomycin sulphate, and 100 units/mL penicillin G (Gibco BRL, UK), at 37°C in a humidified 5% CO_2_/air atmosphere. HeLa cells stably transfected with the human myc-tagged KDELR (HeLa-myc cells) were kindly provided by W. Hsu.

*Transfections*. The cells were transfected with Fugene 6 (Roche, Basel, Switzerland) according to the manufacturer instructions. 

*RNA Interference*. The cells were transfected with Lipofectamine (Thermo Fisher Scientific Inc.), according to manufacturer instructions. PHB siRNA LQ-010530-00 (Dharmacon, Denver, Co., USA). 

*VSV Infection*. The cells were infected with VSV as previously described (Mironov et al., 2001). 

*Transport Pulse Protocols*. The VSVG transport pulses were as previously described (Mironov et al., 2001). Cycloheximide (Sigma Aldrich, WI, USA) was added at 50 *μ*g/mL at the temperature shift.

### 4.3. Protein Analysis

Following the transport protocol, the cells were washed three times with ice-cold PBS and harvested immediately in lysis buffer (1% Triton X-100, 20 mM Tris-HCl, pH 8.0, 150 mM NaCl, 1 mg/mL Na_3_VO_4_, 5 mM PMSF, 5 *μ*g/mL each leupeptin, aprotinin, pepstatin), at 4°C. The cell lysates were centrifuged at 15,000 ×g for 5 min at 4°C, to pellet and remove the nuclei. The postnuclear supernatant was immediately processed for SDS-PAGE and Western blotting. Of note, this separation of the postnuclear supernatant from the nuclei can be crucial for the detection of tyrosine phosphorylation.

### 4.4. Coimmunoprecipitation

To prepare total cell lysates, cells (10^7^/150-mm plate) were cooled on ice, washed with ice-cold 0.9% NaCl (3 times) (Diaco, Italy), scraped, and lysed with 1.8 mL ice-cold lysis buffer (50 mM Tris-HCl, pH 7.4, 150 mM NaCl, 2 mM MgCl_2_, 1 mM EDTA, 1 mM *β*-mercaptoethanol, 1% Triton or 15 mM CHAPS (Sigma Aldrich) and a cocktail of protease inhibitors (Roche)). Subsequently, the total lysates were passed through a syringe needle (15 times) and incubated on a rotating wheel for 1.5 h at 4°C. The lysates were then cleared by centrifugation at 14,000 rpm for 15 min. The supernatants (4–6 mg protein) were incubated overnight with agarose beads coupled to rabbit polyclonal c-myc antibody (50 *μ*L/mg) (Santa Cruz Biotechnology). The settled beads were extensively washed with lysis buffer, and the bound protein was eluted 10 times with 0.1 M ammonium hydroxide, pH 11. The eluted samples were dialysed against 500 mM ammonium hydroxide, pH 11, overnight at 4°C, and then concentrated by lyophilisation.

### 4.5. Two-Dimensional Gel Electrophoresis

The lyophilised immunoprecipitated proteins were resuspended in rehydration buffer (5 M urea, 2 M thiourea, 2% CHAPS, 2% Zwittergent, 40 mM dithiothreitol (Sigma Aldrich), and 1% IPG buffer (Amersham)). Proteins were separated by isoelectric focusing using an IPGphor apparatus (Amersham). In-gel rehydration was carried out on immobilised 13-cm IPG strips with a broad pI range: pH 3–10 linear gradient (Amersham). The optimised isoelectric focusing conditions were 20°C, 50 *μ*A/strip: Step 1, rehydration at 30 V for 12 h; Step 2, gradient to 1000 V for 10 h; Step 3, step-n-hold 1000 V for 1 h; Step 4, gradient 8000 V for 1 h; Step 5, step-n-hold 8000 V for 3 h; for a total of 34360 V/h.

For the second dimension, the IPG strips were equilibrated for 15 min in 6 M urea, 50 mM Tris-HCl, pH 8.8, 30% glycerol, 2% SDS, 1% dithiothreitol, and for 15 min in 6 M urea, 50 mM Tris-HCl, pH 8.8, 30% glycerol, 2% SDS, 100 mM iodoacetamide. Proteins were separated using 12.5% SDS-PAGE, following standard protocols, and revealed with mass-spectrometry compatible silver staining.

### 4.6. Matrix-Assisted Laser Desorption/Ionization Time-of-Flight Mass Spectrometry

Protein bands were excised from the SDS-PAGE and placed in 0.5 mL microcentrifuge tubes (Eppendorf; Hamburg, Germany). After washing, the cysteines were reduced and alkylated with iodoacetamide [46]. The samples were digested with sequencing-grade modified trypsin (Promega, Madison, WI, USA) in 40 mM ammonium bicarbonate at 37°C overnight, under slight shaking on a thermomixer. The reaction was stopped with H_2_O/0.1% trifluoroacetic acid at 30°C, for 15 min. The resulting tryptic peptides were extracted, desalted with ZipTip C_18_ columns (Millipore Corp., Bedford, MA), directly eluted, and crystallised in a saturated solution of *α*-cyano-4-hydroxy-cinnamic acid in 50% (v/v) acetonitrile/H_2_O.

The samples were then spotted onto stainless steel MALDI sample target plates, and the peptide mass spectra were obtained by MALDI TOF MS (Reflex IV; Bruker Daltonics, Bremen, Germany), with a nitrogen laser and an emission wavelength of 337 nm. The mass spectra were acquired in positive-ion reflectron mode with delayed extraction and 20 kV acceleration voltage. External calibration was performed for each measurement, using a mixture of seven standard peptides (average mass accuracy, >20 ppm). All of the mass spectra were acquired using a minimum number of 250 laser shots. The spectra were internally calibrated with trypsin autolysis products. Peptide matching and protein searches were performed through submission of the peptide mass lists to database searches (NCBInr and/or SWISS PROT), using the Mascot and ProFound search engines.

### 4.7. Fluorescence Immunostaining and Confocal Microscopy

The cells were grown to subconfluent density on glass coverslips for 24 h and then washed with PBS. Following fixing in 4% paraformaldehyde (Sigma Aldrich) for 10 min at room temperature, they were incubated in blocking solution (0.05% saponin, 0.5% bovine serum albumin, 50 mM NH_4_Cl; Sigma Aldrich) in PBS for 30 min at room temperature. The cells were subsequently incubated with the specified antibodies diluted in blocking solution, for 2-3 h at room temperature, or overnight at 4°C. After incubation with the primary antibody, the cells were washed three times in PBS and incubated with a fluorescent conjugated anti-IgG secondary antibody for 1 h at room temperature. For the triple labelling (rabbit/mouse/sheep), Alexa 488-conjugated anti-rabbit or anti-mouse antibodies raised in chicken were used (rather than in goat), to avoid possible cross-reactions. The cells were finally examined under a confocal microscope (Zeiss LSM 510; Zeiss, Thornwood, NY, USA). The quantification of fluorescence signals was as follows. The area of interest was delineated manually and the fluorescence intensity was quantified using the LSM510-3.2 software (Zeiss). To assess the colocalization we removed the background immunofluorescence and used the colocalization functions of the LSM510-3.2 software (Zeiss).

## Supplementary Material

Characterization of HeLa-myc cells.
Supplementary Fig S1. shows that the intracellular distribution of myc-tagged KDELR stably transfected in Hela cells (HeLa-myc cells) was similar to that of endogenous KDELR. Also, the Golgi morphology was not affected in the HeLa-myc cells, as assessed by GM130, mannosidase II and TGN46 staining.
Supplementary Fig S2. shows that the transport efficiency of VSVG in HeLa-myc cells was similar to that in wild-type HeLa cells.
Supplementary Fig S3. shows that the β subunit of the COPI coatomer complex coimmunoprecipitated with the KDELR from HeLa-myc cell lysate.
