# Supplementary material for: Prohibitin: A Novel Molecular Player in KDEL Receptor Signalling
Source: Biomed Res Int. 2015 May 10;2015:319454. doi: 10.1155/2015/319454 (PMC4442004; doi:10.1155/2015/319454)

Supplementary Figure legends

Supplementary Figure S1. Morphological characterisation of HeLa-myc cells. Wild-type (HeLa) and HeLa-myc cells were fixed and stained for myc (HeLa-myc cells only; red), endogenous KDELR (green), GM130 (blue; A), mannosidase 2 (MAN II, blue; B), and TGN46 (blue; C); the merged images are also shown. Scale bars, 10 μm.

Supplementary Figure S2. Efficiency of VSVG trafficking in HeLa-myc cells. Wild-type (HeLa) and HeLa-myc cells were transfected with VSV-GFP and incubated overnight at 40 °C (temperature block), and then shifted to 32 °C for the indicated times (temperature-block release). Panels 0 and 30 min at 32 °C: merged images of VSVG-GFP fluorescence and GM130 staining (marker for *cis*-Golgi area; red). Panels 45 min at 32 °C: merged images of VSVG-GFP fluorescence and TGN46 staining (marker for *trans*-Golgi area; red). Panels 60 min at 32 °C: merged images of VSVG-GFP fluorescence and staining of VSVG at the plasma membrane (revealed by an antibody against the extracellular domain of VSVG; External VSVG; red). Scale bars, 10 μm.

Supplementary Figure S3. KDELR coimmunoprecipitates with COPI coatomer. Wild-type HeLa cells and HeLa-myc cells were immunoprecipitated using anti-myc antibodies. The immunoprecipitated proteins were separated by polyacrylamide gel electrophoresis and analysed by Western blotting for KDELR-myc, and β-COP.

Supplementary Figure S1

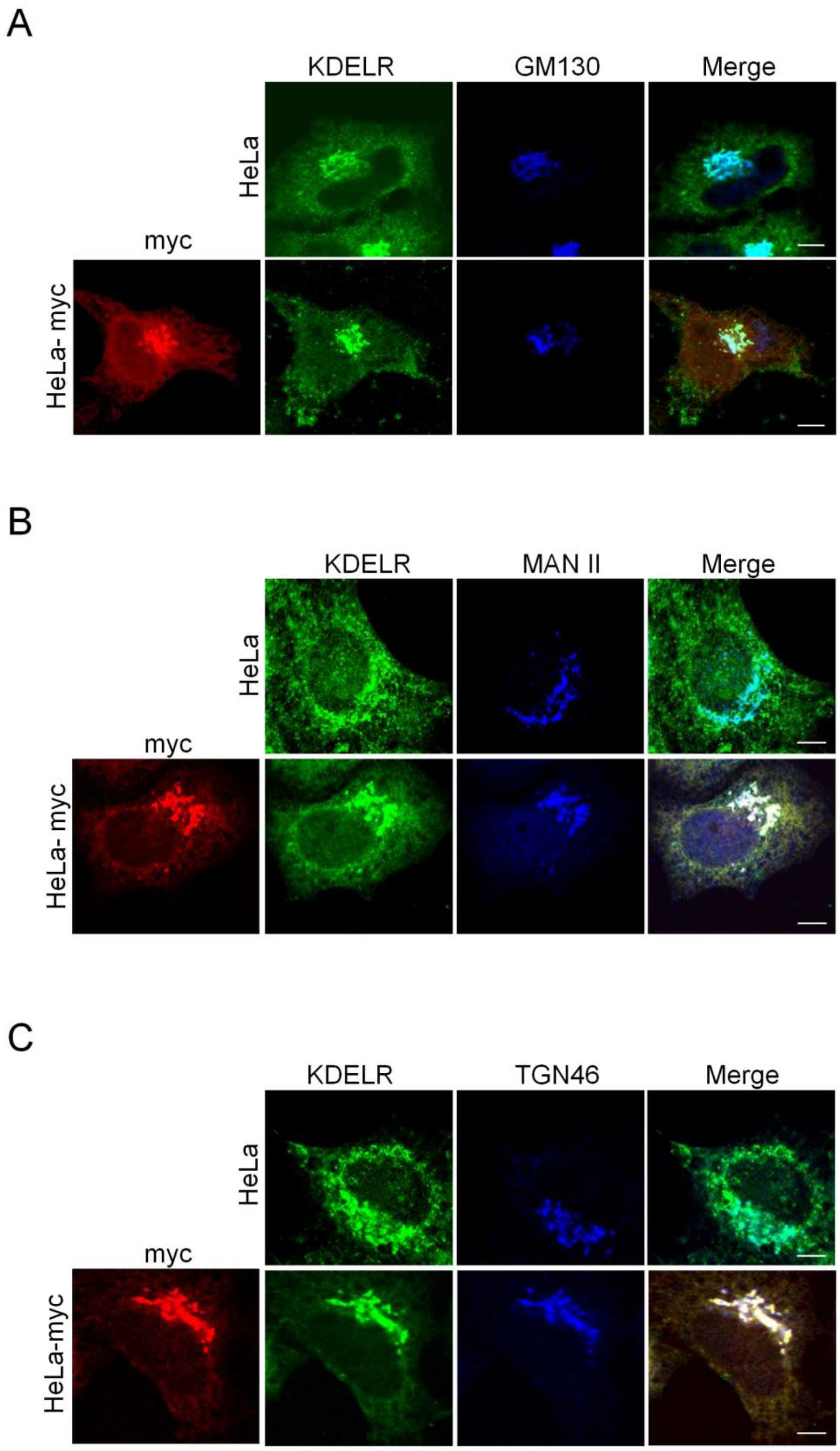

Supplementary Figure S2

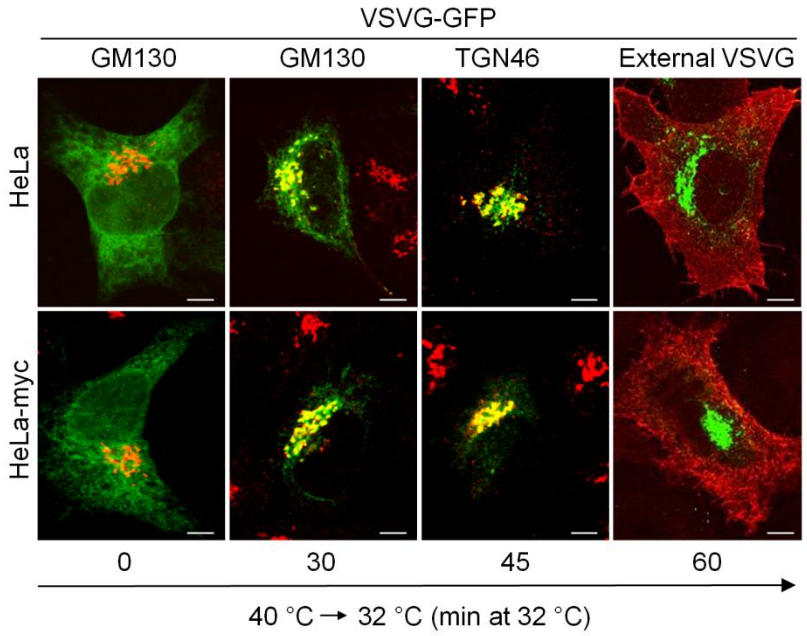

Supplementary Figure S3

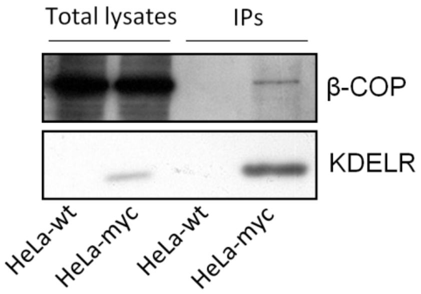

Supplement: Supplementary file 1 — Characterization of HeLa-myc cells. Supplementary Fig S1. shows that the intracellular distribution of myc-tagged KDELR stably transfected in Hela cells (HeLa-myc cells) was similar to that of endogenous KDELR. Also, the Golgi morphology was not affected in the HeLa-myc cells, as assessed by GM130, mannosidase II and TGN46 staining. Supplementary Fig S2. shows that the transport efficiency of VSVG in HeLa-myc cells was similar to that in wild-type HeLa cells. Supplementary Fig S3. shows that the β subunit of the COPI coatomer complex coimmunoprecipitated with the KDELR from HeLa-myc cell lysate. [file 319454.f1.pdf]
